# Decision Making Deficits in Relation to Food Cues Influence Obesity: A Triadic Neural Model of Problematic Eating

**DOI:** 10.3389/fpsyt.2018.00264

**Published:** 2018-06-14

**Authors:** Rui Chen, Danni P. Li, Ofir Turel, Thomas A. Sørensen, Antoine Bechara, Yonghui Li, Qinghua He

**Affiliations:** ^1^Faculty of Psychology, Southwest University, Chongqing, China; ^2^Sino-Danish Center for Education and Research, Beijing, China; ^3^Department of Psychology, University of Chinese Academy of Sciences, Beijing, China; ^4^Center of Functionally Integrative Neuroscience, Institute for Clinical Medicine, Aarhus University, Aarhus, Denmark; ^5^College of Business and Economics, California State University, Fullerton, Fullerton, CA, United States; ^6^Department of Psychology, University of Southern California, Los Angeles, CA, United States; ^7^Department of Communication and Psychology, Centre for Cognitive Neuroscience, Aalborg University, Aalborg, Denmark; ^8^Institute of Psychology, Chinese Academy of Sciences, Beijing, China; ^9^Chongqing Collaborative Innovation Center for Brain Science, Chongqing, China

**Keywords:** obesity, impulsive system, reflective system, interoceptive system, tripartite model of addictive behaviors, prefrontal cortex, insular cortex, amygdala-striatum system

## Abstract

In this review article we propose a model of the brain systems, the deficiency of which may underlie problematic eating. This integrative model is based on studies that have focused on discrete brain components involved in problematic eating, combined with insights from studies on the neurocognitive basis of other addictive and problematic behaviors. The model includes: (a) a hyper-functioning reward anticipation and processing system (amygdala-striatum dependent) in response to food-related cues; (b) a hypo-functioning reflective and inhibitory control system (prefrontal cortex dependent), that fails to anticipate and properly weigh future outcomes; and (c) an altered interoceptive awareness system (insular cortex dependent) that translates homeostatic violation signals into a strong consumption desire that hijacks the inhibitory system and excites the reward system. We posit that when the abovementioned systems are imbalanced in such a way that the dopamine axis is hyperactive in relation to food cues and the inhibitory system is weak, and this is further aggravated by an altered interoceptive awareness system, people may experience loss of control or inability to resist tempting/rewarding foods. This loss of control over food consumption can explain, at least in part, the development of excess weight and contribute to the obesity epidemic.

## Introduction

Obesity (BMI = kg·m^−2^ > 30) is one of the most serious health issues in the developed world; it affects approximately 500 million people (1). In high-income English-speaking countries, 70% of the population were overweight (BMI > 25) or obese in 2014. In China, the absolute number of obese individuals has now surpassed the number in the United States, making China the most obese country in the world (2). Obesity and overweight are major risk factors for developing multiple lifestyle diseases such as hypertension, type II diabetes mellitus, metabolic syndrome, and cardiovascular diseases (3). They therefore have major financial implications for people and nations (1).

Obesity can be viewed as a chronical lifestyle disorder caused by overconsumption of calories relative to calorie expenditure. It is a multifactorial problem affected by environment, culture, socio-economic status, and genetics (4). Recent research has indicated that obesity may be, at least partially, rooted in decision-making deficits in relation to food; and that such deficits resemble those observed in other addictive and problematic behaviors. That is, a strong “wanting” of food, combined with poor inhibition and foresight abilities, can explain why some people overconsume food. The terms “food addiction” and/or “eating addiction” encapsulate this perspective and imply that the rewarding and reinforcing properties of foods combined with the way humans decide on food consumption, make excessive food consumption similar to the consumption of rewarding substances, or to the enactment of other addictive behaviors (5–10).

In support of this perspective, animal studies have demonstrated that there are similarities between the neural mechanisms that underlie substance addiction and excess food consumption. For example, Johnson and Kenny (11) showed that highly palatable and processed foods may trigger biological changes in the mesolimbic pathways (part of the reward system) of obese rats. Striatal dopamine D2-receptors were downregulated in obese rats compared to in their lean counterparts, thereby triggering compulsive eating behavior and addiction-like neuro-adaptive responses.

Neuroimaging studies in humans have also provided evidence for similarities between substance and behavioral addictions and excess food consumption. Volkow et al. (12) reviewed the overlapping neural circuits in addiction and obesity; they showed that both drugs and palatable food can act on reward, motivation, learning, and inhibitory control systems. In further support of this view, a meta-analysis by Garcíagarcía et al. (13) included 87 studies and mapped the blood oxygen level-dependent (BOLD) functional magnetic resonance imaging (fMRI) response to reward in participants with obesity, substance addiction and non-substance addiction, including pathological gambling and Internet gaming. They observed various overlaps between obesity and substance addictions in BOLD activations, but fewer commonalities between non-substance related addictions and obesity were found. These results indicate that it is likely that at least some obese individuals can share similar neurological and physiological impairments in the reward circuitry of their brains with substance addicts.

Due to the possible similarities between substance and behavioral addictions and excess food consumption, we propose a triadic neurocognitive model of excess food consumption and the resultant obesity. This model is based on research from the substance addiction domain combined with anecdotal evidence regarding brain systems that can underlie excess food intake and obesity.

## The triadic neurocognitive system

Individuals suffering from addictive disorders are characterized by compulsive drug- or behavior-seeking conduct, despite facing negative consequences such as financial and emotional problems when they continue with substance-consumption or addictive-behavior enactment. These addictive behaviors are often viewed as rooted in impaired behavioral learning processes, strong and uncontrollable impulsions, weak self-regulation, and impaired decision-making abilities (14–18). These impairments reflect a simplistic dual system view of addictions, which portrays the imbalance between a hyperactive bottom-up impulsive reward system and a hypoactive top-down reflective control system as the decision-making deficit that subserves over consumption of substances and enactment of behaviors (19). However, recently a more refined hypothesis has been proposed, that addictions are also subserved by a disrupted insula-mediated interoceptive awareness system (20, 21). The impulsive and reflective systems together reflect a dual-process view, in which one (the impulsive system) is faster, autonomic, and subconscious, and the other (the reflective system) is slower, deliberative, and conscious (22, 23). Adding the interoceptive awareness system creates a triadic model. This interoceptive awareness system integrates homeostatic signals, thereby regulating processes of the dual-process system (24) and subserving behaviors through the subjective feelings of urges /cravings that it mediates (25). Evidence has been accumulating regarding the viability of the triadic system perspective for explaining excessive behaviors (26, 27), as well as regarding the role of the interoceptive awareness system in decisional deficits (28)

If indeed food overconsumption can be viewed as a decision making deficit that is similar to substance addictions in that it is rooted in an imbalance between the abovementioned three systems, then a tripartite model of decision making in relation to food cues can explain the loss of control or inability to resist tempting/rewarding foods (and the resultant obesity). Based on this perspective, food-related stimuli can trigger bottom-up involuntary habitual desire mediated by the amygdala-striatal system, the goal-driven reflective system can fail to anticipate future outcomes of food overconsumption and/or fail to inhibit excess food intake, and this imbalance can be exacerbated by an altered interoceptive awareness system that hijacks inhibition/reflection resources and excites the impulsive system.

**Figure 1:**
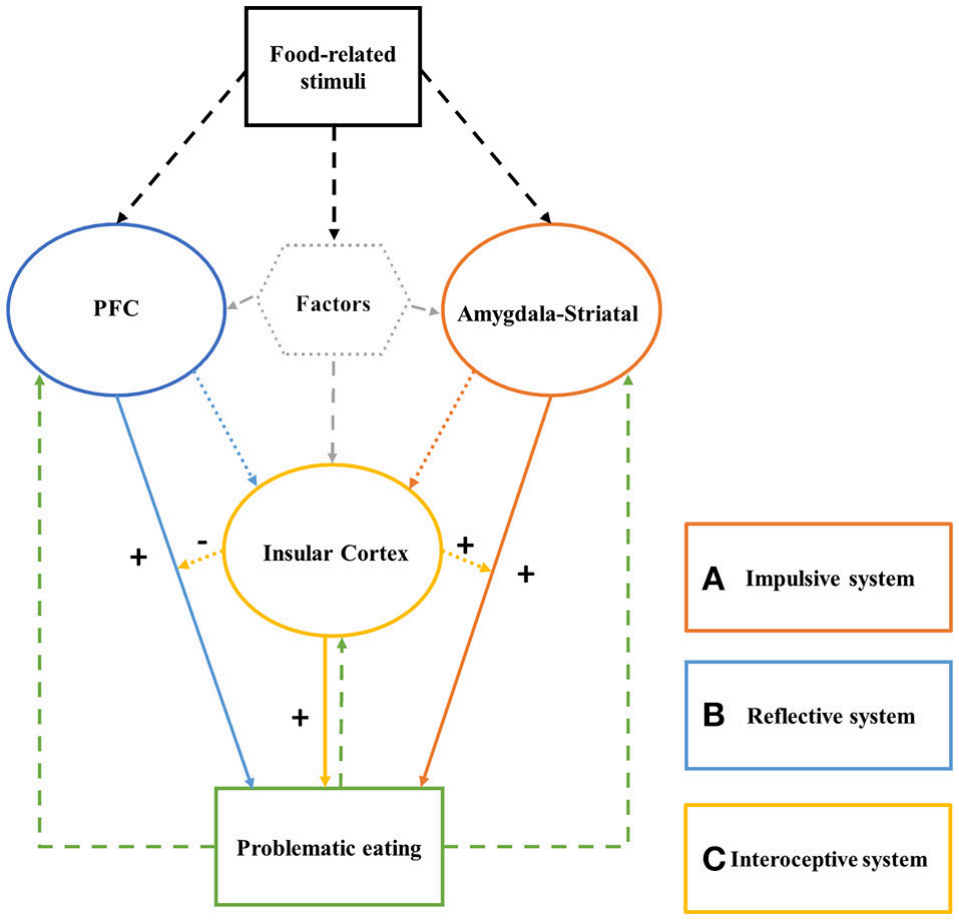
The Triadic System of problematic eating consists of: (a) a hyper-functioning impulsive system; (b) a hypo-functioning reflective/inhibition system; and (c) an altered interoceptive awareness system. After exposure to food-related stimuli, the impulsive system generates fast, automatic, unconscious, motivation to consume the food. The reflective system, if intact, considers future outcomes of this act, and inhibits it as needed. The interoceptive awareness system acts as a modulator of the impulsive and reflective system effects; its activation excites the impulsive system and “hijacks” the cognitive resources the reflective system requires for reflecting on and inhibiting food consumption.

The aim of this mini-review is to provide initial support for this perspective. To do so, we integrate evidence regarding an impaired triadic system in individuals with problematic or disordered eating. Our proposed neurocognitive model of problematic eating is depicted in Figure 1. We suggest that the portrayed imbalance can provide one reasonable explanation for excess food intake.

### The impulsive system

The development and maintenance of addictions can be explained via the incentive salience theory, which couples classical Pavlovian conditioning principles (29, 30) with associative learning mechanisms by automatically generating drug- or gambling-related action and craving (19). Environmental cues that trigger such responses are mediated by subcortical structures of the basal ganglia and its cortical inputs. Specifically, the dopamine-dependent striatum-amygdala neural circuit promotes habitual, incentive-motivational and salient behaviors in response to non-natural rewards [e.g., psychoactive drugs; see (31)], natural rewards (e.g., food, and sexual intercourse) (32) and behavioral cues (33–36).

Addictive substances, including highly palatable foods, can sensitize vulnerable reward systems to food stimuli and lead to hyper–activation. Eating can lead to the release of dopamine in the ventral tegmental area (VTA) and its transmission to the nucleus accumbens (NAcc; a part of the ventral striatum), which in turn, translates it into release of opioid peptides that make people feel good. Learning to expect and subconsciously anticipate such rewards from food consumption induces in some people strong motivational states for food intake, which, if remains unchecked or uninhibited, can drive excess eating (37).

Indeed, increased activation in the amygdala-striatal area has been reported in many fMRI studies that used food-related stimuli. For example, obese individuals had greater activation in brain areas associated with reward processing when exposed to visual food cues compared to lean individuals, but without food stimulation this activation was decreased 38–40. Morris and Dolan (41) reported a PET study according to which the activation of the left amygdala and the right anterior orbitofrontal cortex (OFC) correlated positively with recognition memory for food items and the activation of the right OFC was negatively correlated with recognition memory for non-food items. Ng et al. (42) found that compared to lean people, obese people show higher activity in somatosensory (Rolandic operculum), gustatory (frontal operculum), and reward valuation (amygdala) regions, and in the ventromedial prefrontal cortex (vmPFC) in response to intake and anticipated intake of a milkshake vs. a plain drink. Similarly, He et al. (43) used a food-specific go/nogo task to measure the inhibitory control ability of high BMI participants. Results indicated that high BMI participants responded faster in go trials related to high-calorie food images, compared to the go trials related to low-calorie food images. In nogo trial, these participants expressed stronger difficulty to inhibit their responses. In addition, fMRI results showed that the right striatum was more active in go trials focusing on high-calorie food images, and that the PFC was more active in nogo trials; PFC activation negatively correlated with subjects' BMI. Contrerasrodríguez et al. (44) examined potential abnormal functional connectivity in obese people. They found that over-weight participants displayed increased functional connection between the ventral striatum and the medial prefrontal and parietal cortices; and between the dorsal striatum and the somatosensory cortex. Moreover, these participants' connectivity of the dorsal striatum was associated with food craving and can predict BMI increases. These results support the view that a hyper-responsivity of the impulsive system (and a deficit in the reflective system) may promote excess, impulsive eating behaviors.

Likewise, Coveleskie et al. (45) have examined the structural/anatomical differences between obese women and lean controls. Voxel-Based Morphometry (VBM) analysis revealed significantly greater gray matter volume (GMV) in the NAcc in the high BMI-group compared to the control group. This suggests that brain structures, and specifically increased GMV in the areas associated with the impulsive reward system, may be a marker for obesity and essentially food “addiction.”

Collectively, such studies indicate that the impulsive system likely plays an important role in processes that automatically gauge the value of food-related stimuli and generates motivational consumption states. This motivational state, if not assessed and inhibited properly, may result in problematic eating.

### The reflective system

The impulsive system is indeed a key mediator of the “wanting” component during reward anticipation and processing; it creates strong incentive-motivation to act. However, it does not account for controlling the “wanting” and acting upon it. These are regulated by the reflective system, which includes vmPFC, anterior cingulate cortex, dlPFC, and lateral orbitofrontal/inferior frontal gyrus (32). The PFC is involved in decision-making, executive functions, forecasting flexible future outcomes, and controlling the action drives mediated via the impulsive system (46). Indeed, damage to this area impairs decision-making abilities; it results in reckless decisions without sufficient consideration of the consequences of the behavior (47).

The reflective system is based on the integration of two functions that are mediated via two neural sub-systems; “cool” executive functions and “hot” executive functions (48). “Cool” executive functions are mediated via the dorsolateral prefrontal cortex (dlPFC) and anterior cingulate cortex (ACC), and involve inhibitory control, basic working memory, analytical thinking, cognitive flexibility, and the maintenance and updating of new relevant information (49). This view is in congruence with theories of economic decision-making, according to which humans are rational, reflective, and goal-directed decision-makers. In contrast, “Hot” executive functions integrate emotions into decision making, and are mediated by the ventromedial PFC (vmPFC) and the OFC. These structures are involved in eliciting somatic states from memories and knowledge, which in return initiate emotional and affective responses (50), and consequently are based on one's “gut-feeling,” intuition, and heuristics (51). Adequate decision-making often involves an integration of the “cool” cognitive and “hot” emotion-involved systems; it relies on one's ability to more optimally evaluate probable outcomes of an action by weighing short-term gains against long-term, often more uncertain, losses (52, 53).

A growing body of fMRI studies has examined the abnormal function of the reflective system in obese people. Le et al. (54) compared the activation of the left dlPFC in lean and obese people following a meal. They discovered lower activity in obese people. A meta-analysis conducted by (55) examined the most common functional differences between normal-weight and obese people responding to food stimuli. The results indicated that obese participants had increased activation in the left dorsomedial prefrontal cortex, right parahippocampal gyrus, right precentral gyrus, and right anterior cingulate cortex, as well as reduced activation in the left dlPFC and left insular cortex. This decreased activation may be indicative of the notion that obese people have weaker inhibitory control and interoceptive awareness when exposed to food-related stimuli, compared to people in the normal weight range. Combining such inhibitory deficits with high sensitivity to food stimuli, often presented by obese people, can provide a neurocognitive account for problematic eating and consequent obesity.

An fMRI study investigating unhealthy food choices (43) found that increased activity in the PFC and reduced activity of the insula were positively correlated with high consumption of vegetables. In contrast, consumption of high calorie foods correlated with reduced activity of the PFC and elevated activity of the insula. These results indicated that in order to make healthy food choices, people have to spend more cognitive resources encapsulated in the reflective system. Moreover, Verdejo-Román et al. (56) found less effective functional connectivity between frontal areas responsible for cognitive control and striatal areas involved in reward anticipation and processing in obese individuals who performed a food-based reward task. This suggests that the reward circuitry of obese people is incongruent with the cognitive control areas, thus making obese individuals' self-regulation ability poor when trying to control consumption of palatable foods.

Structural imaging studies using VBM also support this perspective (57, 58). Specifically, a longitudinal study (59) examined a total of 292 obese and normal weight subjects over two time-points, 5 years apart. Obese subjects had significantly smaller total brain volumes (with no difference in white matter; or cerebrospinal fluid). The most robust finding in their study was the reduced GMV in dlPFC in obese people, which can be indicative of poor foresight and risk assessment, and is common in other addictions (60).

Taken together, these studies indicate that obese individuals may have a hypo-functioning reflective system, which can manifest structurally through decreased GMV in key regions of the reflective system. This again points to possible similarities between problematic eating and other addictive behaviors.

### The interoceptive-awareness system

The third and final component in the tripartite model is an interoceptive awareness system, which is primarily insular cortex dependent. This system is reciprocally connected to several limbic regions including the vmPFC, the amygdala, and the ventral striatum (61). Besides being the primary taste area in the brain (4), it has recently been argued that the insular cortex may contribute to the onset and maintenance of addiction by integrating and translating interoceptive, somatic signals into subjective experiences like the feeling of anticipation, desires, urges, or cravings (53). These interoceptive manifestations can potentiate the activity of the impulsive system, and simultaneously weaken the goal-driven cognitive functions mediated by the reflective system. In other words, interoceptive signals can “hijack” the cognitive resources of the reflective system that are required for executing inhibitory control over tempting behaviors, such as eating high-calorie dense foods.

This view is supported by fMRI studies showing increased insular activity in response to food-related stimuli. According to Simmons et al. (62) and Frank et al. (63), when presented with high calorie pictures of food, subjects show increased activity in the insular cortex and the OFC. In a recent review article (64), neural responses to visual food cues according to weight status were analyzed. Findings indicated that both the insula and OFC have increased activity in obese subjects in a majority of the sixty fMRI studies they reviewed. In addition, increased brain response to appetitive tastes in both the insula and the amygdala have been demonstrated in satiated obese compared with satiated healthy weight children (65). Deficient emotion regulation can also be associated with obesity. Steward et al. (66) asked participants to modulate their negative emotions induced by negative pictures. Overweight participants continuously displayed high activation in the right insula. Functional connectivity between the right insula and right dorsal lateral PFC and dorsal medial PFC was less effective in overweigh participants compared to normal-weight participants.

Studies of adolescent obesity indicated that the activation of the insula was positively correlated with interoceptive sensitivity in obese adolescents, but negatively correlated with interoceptive sensitivity in healthy-weight adolescents. While in healthy weight adolescents insular activation negatively correlated with external eating, it positively correlated with external eating in obese adolescents (67). Although the insula activated in both healthy and overweight adolescents, it reflected different mechanisms in these groups. The interoceptive insensitivity in obese people may explain increased food consumption; this consumption serves as a means to achieve equal satiation to this experienced by normal weight individuals.

VBM studies also support the hypothesis that obese or individuals with disordered eating have similar structural differences in the GMV and white matter in the insular cortex as substance addicts. Both Shott et al. (68) and Jauchchara et al. (69) studied gray and white matter densities in obese cases vs. lean controls. They both found that areas associated with the impulsive system (striatum and putamen) and the interoceptive awareness system (the insula) had reduced GMV [and associated white matter in (68)] in obese subjects. Smucny et al. (70) however, have shown that the reduction in insular GMV also can be seen in lower ranges of BMI. After controlling for age, sex, and total GMV, the results showed that obesity-prone people had reduced GMV in the OFC and insula compared to obesity-resistant subjects. They also found that GM volume in the insula was inversely correlated with reported ratings of hunger after a meal and inversely correlated with plasma leptin concentration. These findings illuminate the importance of and associations between GMV, homeostatic and hormonal responses, as related to food intake.

Other than obesity, studies also showed that the following factors can be associated with differences in the activity of the insula; and indirectly influence impulsive and reflective system functions.

#### An eating disorders factor

Brain imaging studies showed that abnormal activity of the insula exists in different eating disorders. When presented with food stimuli, the OFC, ACC and insula exhibited increased activation in all participants. However, eating disorder patients showed stronger medial OFC activity, whereas bulimic patients presented greater activation of both the ACC and the insula (71). Furthermore, Kim et al. (72) measured the functional connectivity of the anterior insula in both anorexia nervosa (AN) and bulimia nervosa (BN) and reported that the left anterior insula was significantly connected with the right insula and right inferior frontal gyrus in the AN group. Nevertheless, in the BN group, the left anterior insula showed significant connection with the medial OFC.

VBM studies examining recovered AN and BN patients (who had to be symptom-free for a minimum of 2 years) (73) found that recovered patients had increased insular GMV volume compared to women without any history of eating disorders. However, a study investigating WM in actual BN patients found decreased fractional anisotropy in the bilateral corona radiata extending into the posterior limb of the internal capsule, corpus callosum, and right sub-insula (74). These results collectively underline the differences within the spectrum of disordered eating; and more importantly emphasize the possible association between diet and eating behavior's and the GMV in the insula and insula-associated areas.

#### Satiation and deprivation factors

The distinction between different somatic states, specifically hunger vs. fullness, also seem to be an important factor for insular activity. A meta-analysis including ten fMRI studies examining the non-fasted neural response to food stimuli in obese vs. lean subjects found significant evidence for reduced activation in the left dlPFC and the left insular cortex (55). However, it has also been suggested that food cue presentation can significantly increase activation in superior temporal, anterior insula, and orbitofrontal cortices during a fasting state (75), and that increased activation of OFC was positively correlated with ratings of hunger. Moreover, Uher et al. (76) found that the response to taste stimuli in the left anterior insula was significantly stronger during a fasting state compared to in the satiated state in healthy normal weight individuals. Similarly, healthy satiated females demonstrated increased activity in both lateral and medial OFC, PCC, caudate, putamen, fusiform gyrus, and the insula when responding to low calorie food cues. In contrast, activation during a deprivation state was related to reward processing areas increases following the presentation of high calorie food cues (77).

Thus, it may be especially important to acknowledge differences in insular activity during deprivation vs. satiation, regardless of the type of the deficient decision-making context (e.g., drugs, food, addictive behaviors). In line with these findings, recent result indicated that the reflective system (Specifically dlPFC and ACC) was engaged when participants tried to control food-related responses in an fMRI food-specific go/nogo task (He et al., under review). It was also found that the vmPFC was activated in inhibitory control attempts executed when the participants were satiated. In addition, these results revealed that the insular cortex was significantly more active during deprivation vs. during satiation states. Importantly, these changes were more pronounced in participants with higher BMI compared to their lean counterparts. Hence obese individuals may be hypersensitive to interoceptive/somatic signals of hunger, following an increased positive alliesthesia (the affective part of sensation), incentive-salience and craving for food cues, as well as being insensitive to interoceptive/somatic signals of satiation (78). Collectively these studies indicate that deprivation vs. satiation can modulate insular activity in all people, but especially among obese individuals.

## Conclusion and future directions

The proposed triadic neurocognitive model of problematic eating includes three systems. First, the impulsive system contains the amygdala-striatal circuits. It processes reward expectations and production in response to food-related cues and/or food consumption. Second, the reflective system includes the PFC and its sub-regions. It mediates planning, anticipation of future outcomes and behavior inhibition processes. Lastly, the interoceptive awareness system includes the insula. This system translates homeostatic and interoceptive signals into self-awareness regarding desires/urges/cravings; presumably also in relation to food cues and satiation/hunger states.

As individuals develop social expectations and norms, as well as the ability to set long-term goals, the reflective system can inhibit the action motivation generated via the impulsive system if this action is deemed as sub-optimal. When the systems are balanced, there are no major decision making deficits beyond typical biases [e.g., social desirability bias, framing bias, sunk cost bias, etc.; see Uher et al. (79)]. However, when the impulsive system is hyperactive and the reflective system is hypoactive, the behavior, in our case food consumption, becomes impulsive, unplanned, disadvantageous, disordered, and problematic. The interoceptive awareness system extends this view, because it can occupy the reflective system and suppress the inhibitory control function, and simultaneously further excite the impulsive system. Under such circumstance, the existing imbalance or gap between the strength of the impulsive and reflective systems widens, and the behavior patterns becomes much more impulse-driven (26). Together, these decision making deficits can influence over-consumption of food and ultimately obesity.

Although in this article we portray a triadic model of problematic eating, which may influence obesity based on addiction research, it is critical to consider the fact that individuals with problematic eating can be neurologically different from individuals with substance addiction. Moreover, in comparison to drug cues, cues associated with food are multimodal and less salient in terms of their interoceptive effects. Palatable foods may begin as relatively strong reinforcers compared to drugs, but cocaine and other drugs create implicit associations that last longer than associations between stimuli paired with natural reinforcers such as food (80, 81). Hence, despite the many similarities between substance and food consumption, more research is needed to shed light also on possible differences between neural deficits that may subserve excessive food and substances intake.

This need for more research is exacerbated by the fact that the intersection between neural mechanisms of the proposed triadic model and excess food consumption is relatively new, yet has the potential to produce strong theoretical and practical implications. For example, understanding the neural systems sub-serving excess eating can lead to pharmacological- or cognitive-behavioral therapies that may help excess eaters to overcome their food indulgence. Moreover, such future research is also of social importance, because it would potentially help society to understand how and why some individuals are more vulnerable to environmental food related cues, not simply from a conventional dietary perspective, but also from a decision-making standpoint. We hence call for more research on obesity from a neurocognitive decision making perspective. Last, the proposed neurocognitive model still requires empirical support and possible fine-tuning, because physiological aspects can also play an important part of weight management, and hormones can impact interoception of satiety and hunger. Such aspects should be included in extension of the model proposed here; and we call for future research to account for such extensions.

## Author contributions

OT, TS, AB, YL, and QH conceived the model and study structure. RC and DL wrote the first draft. OT, TS, AB, YL, and QH made critical revisions. All authors approved the final version of this manuscript.

### Conflict of interest statement

The authors declare that the research was conducted in the absence of any commercial or financial relationships that could be construed as a potential conflict of interest.
